# Review of "Medical Entomology for Students" by M.W. Service

**DOI:** 10.1186/1756-3305-1-12

**Published:** 2008-05-21

**Authors:** Richard D Ward

**Affiliations:** 1 School of Life Sciences, Keele University, Staffordshire, ST5 5BG, UK

## Review

This twelve year-old publication was first in the book stores two years after I took up my own Chair of Medical Entomology at Keele University. It is one of those "first call" volumes off the shelf when you are struggling to remember if endemic or epidemic typhus is transmitted by the tropical rat flea. As an introductory text book for students at undergraduate and postgraduate level, it remains competitively priced, having risen in cost by only £2.99 in four years. In his preface Mike Service points out that the approach has not changed (consisting of morphology – lifecycle – medical importance and control) and that the main areas where changes have been made relate to vector control. Striking new features of the book include a colourful front cover bearing a female *Anopheles farauti *looking replete following lunch and a central block of 24 colour plates depicting a range of vectors of medical importance.

Unlike Lady Thatcher, Mike is not afraid of the odd U-turn and has decided to unadopt the Culicine genus *Ochlerotatus *which is again included as a subspecies of *Aedes*. Many a late night pre-exam revision student will thank him for this kindness. Two useful epidemiological cartoons dealing with the mosquito borne transmission of West Nile Virus and Japanese Encephalitis are added to those on Yellow Fever. As a teaching tool and an *aide mémoire *such graphic summaries are popular with students and an excellent way of clarifying what can verbally appear more complex than it really is. Vector control is a moving canvas with new techniques and products appearing all the time, some stand the test of time making a major impact whilst others have niche roles, are short lived, or remain the dreams of idealistic academics. Dipping in and out of the twenty chapters like an insectivorous bird, I compiled the following assortment of new snippets of information which Mike has added to the book.

A predaceous crustacean called *Mesocyclops *used for biological control of *Aedes *mosquitoes has been used in experimental trials in South-East Asia though its impact is concluded to be very localised. However, the mosquito-eating fish *Poecilia reticulata *is now making a significant impact on malaria in Karnataka India. On genetic control, the author mentions the attempts to engineer mosquitoes unable to transmit pathogens and on a note of caution, points out that the logistics of this approach and its ethics are currently the subject of vigorous debate. On the filariasis front in India and Zanzibar, the use of polystyrene beads to cover larval breeding sites against *Culex quinquefasciutus *continues to be a useful innovation with prevention of disease resurgence when employed following mass drug treatment. On a less optimistic note, the organophosphate Temephos used for *Aedes *control and considered safe for ingestion is now under suspicion of toxigenic and mutagenic effects. Similarly, it is pointed out that insect growth regulators (IGRs) often used in mosquito control may long term be limited in usage since resistance has been observed in both agricultural pests and the blow fly *Lucilia cuprina*. New areas in repellency of insects are noted with the marketing of para-menthane 3,8-diol (PMD) a derivative of lemon eucalyptus oil which is proving to be almost as good as the familiar DEET. Developments in the insecticide impregnated bed net sector are cited, with permanents now being made with deltamethin attached to fibres by resins and re-treatment kits available for domestic application. In China, *circa *38 million Permanets have been distributed as an adjunct during measles vaccination campaigns. The sand fly systematists have been busy, with a reported increase in described species from 600 to nearly 1000 in four years. In addition, there is reference to the successful use of insecticidal impregnated dog collars to control leishmaniasis in Italy and Iran, although no comment is made on the sustainability issue of this system in a country like Brazil, where Feral dogs are a real problem.

The practice of maggot therapy has now been adopted (2005) by at least 20 countries; this involves placing fly larvae on a necrotic wound which they clean very efficiently by consuming dead tissue, a clinical procedure not suited for the squeamish. Last but not least, young children can rejoice in the fact that there is a new non-insecticidal head lice lotion, Hedrin, on the market, which gift-wraps each insect in a 4% solution of dimeticone a silicone that appears to effectively suffocate each of the pests. Future development of resistance is not a strong possibility and dimeticone usage is apparently safe since it is already a common component in cosmetics.

In conclusion, *Medical Entomology for Students *is a "must have" for those studying the discipline and a continuing strength is its frequent updating cycle and the helpful addition and revision of the further reading section.

## Competing interests

The author declares that they have no competing interests.

